# Beta-Blocker Therapy Preserves Normal Splenic T-Lymphocyte Numbers Reduced in Proportion to Sepsis Severity in a Sepsis Model

**DOI:** 10.1155/2019/8157482

**Published:** 2019-12-11

**Authors:** Takeshi Suzuki, Kei Inoue, Toru Igarashi, Jungo Kato, Hiromasa Nagata, Takashige Yamada, Shizuka Minamishima, Hiroshi Morisaki

**Affiliations:** ^1^Tokai University School of Medicine, Department of Anesthesiology, 143 Shimokasuya, Isehara, Kanagawa 259-1193, Japan; ^2^Keio University School of Medicine, Department of Anesthesiology, 35 Shinanomachi, Shinjuku-ku, Tokyo 160-8582, Japan

## Abstract

Lymphocyte cell death contributes to sepsis-induced immunosuppression, leading to poor prognosis. This study examined whether sepsis severity and beta-blocker therapy could affect the degree of T-lymphocyte cell death in a mouse model of sepsis. In the first control study, 20 animals were allocated to 4 groups: control group with sham operation (group C, *n* = 5) and 3 groups with cecum ligation and puncture (CLP) performed at 3 different sites: proximal, middle, and distal cecum (groups CLP-P, CLP-M, and CLP-D, respectively; *n* = 5 in each group). Their spleens were resected under general anesthesia 24 hours after CLP, and the total number of normal splenic T lymphocytes per mouse and the percentage of apoptotic T lymphocytes were evaluated using flow cytometry. In the second experimental study, the effect of the beta-blocker esmolol was examined in CLP-P (group CLP-PE vs. CLP-P; *n* = 5 in each group). The total normal splenic T-lymphocyte numbers per mouse significantly decreased in proportion to CLP severity (group C, 18.6 × 10^6^ (15 × 10^6^–23.6 × 10^6^); CLP-D, 9.2 × 10^6^ (8.8 × 10^6^–9.8 × 10^6^); CLP-M, 6.7 × 10^6^ (6.3 × 10^6^–7.0 × 10^6^); and CLP-P, 5.3 × 10^6^ (5.1 × 10^6^–6.8 × 10^6^)). Beta-blocker therapy restored T-lymphocyte numbers (group CLP-PE vs. CLP-P; 6.94 ± 1.52 × 10^6^ vs. 4.18 ± 1.71 × 10^6^; *p*=0.027) without affecting apoptosis percentage. Beta-blocker therapy might improve sepsis-induced immunosuppression via normal splenic T-lymphocyte preservation.

## 1. Introduction

Sepsis, a life-threatening organ dysfunction caused by a dysregulated host immune response to infection, is one of the leading causes of death in critically ill patients, despite improved medical care and therapeutic approaches. Sepsis is characterized as a hyperinflammatory state that occurs due to neutrophil hyperactivation and inflammatory cytokine overproduction, followed by an immune-suppressive state. During the late sepsis-induced immunosuppressive state, lymphocyte apoptosis has been shown to contribute to immune paralysis, leading to a poor prognosis [1–3]. Thus, to improve the prognosis of septic patients, this late phase must be prevented. Previous experiments have shown that therapies that prevent lymphocyte apoptosis, improve survival in sepsis. However, these therapies have not been clinically tested. Therapies in these studies include caspase inhibitor [4], cell-permeable peptide [5], and siRNA [6] and are unavailable for humans. Thus, there are no lymphocyte apoptosis prevention therapies in clinical use.

Sepsis-induced lymphocyte apoptosis can be caused by various mechanisms, including sympathetic nervous system hyperactivation, inflammatory cytokine overproduction, and reactive oxygen species-induced oxidative stress [3, 7, 8]. Beta-blockers, which can suppress beta-adrenergic stimulation, have been shown to suppress inflammatory cytokine production [9, 10] and attenuate organ dysfunction, including the heart [9], lung [10], and intestine [11], in experimental sepsis models. These beneficial effects of beta-blockers to sepsis, such as suppression of activated sympathetic nervous system and reduction of inflammatory cytokine release, might have an ability to prevent lymphocyte apoptosis and cell death induced by septic insult. Hence, modulation of T-lymphocyte apoptosis and cell death by beta-blocker therapy was examined in a mouse model of sepsis.

## 2. Materials and Methods

This study was approved by the Animal Care and Use Committee of Keio University School of Medicine and was in accordance with the National Institute of Health's guidelines.

### 2.1. Study Protocol

After purchase, male mice (C57BL6, 8–10 weeks, 25–30 g) were acclimatized for 3–7 days in the laboratory and used. They were fed standard rat chow and water ad libitum in a 12 hour light-dark cycle. In this study, only male mice were used since health conditions of male mice are stable in the all seasons compared with female mice.

### 2.2. The First Control Study

Twenty mice were allocated to 4 groups: control group with sham operation (group C, *n* = 5) and 3 groups with cecum ligation and puncture (CLP) performed at 3 different sites: proximal, middle, and distal of cecum (groups CLP-P, CLP-M, and CLP-D, respectively; *n* = 5 in each group). All preparations were performed under general anesthesia. Under sevoflurane anesthesia in oxygen, laparotomies were performed, and ligatures were placed around the cecum at 3 different sites: the cecal quarter site proximal to the ileocecal valve (group-P), midcecum (group-M), and cecal quarter site distal to the ileocecal valve (group-D). A previous study showed that the ligation site could affect sepsis severity. Sepsis was more severe in group-P than group-D [12]. Antibiotics were not given in this study considering the immunomodulatory effects of antibiotics. T lymphocytes were extracted from the spleen, resected 24 hours after surgical preparation, by using the nylon wool column method, and the total number of normal splenocyte T lymphocytes per mouse and the percentage of apoptotic T lymphocytes were evaluated. In brief, fresh spleen was macerated on a 40 *μ*m cell strainer placed on a 50 ml tube with a syringe plunger. The strainer was rinsed with 10 ml of RPMI media to flush splenocytes into the 50 ml tube, and cells pelleted at 400 g for 5 minutes after incubation with 1 ml of ACK lysing buffer to remove red blood cells. The pelleted cells, including splenocytes, were resuspended in RPMI media, added into the nylon wool column, and incubated for 1 hour at room temperature. The nylon wool column was eluted using RPMI media into a 50 ml tube to collect the nonadherent CD3+ T-cell fraction. The resulting cell population is routinely 80–90% CD3+ T lymphocytes. After counting the T lymphocytes per spleen by electron microscopy, T-lymphocyte apoptosis was evaluated using flow cytometry. Pelleted cells were incubated with anti-CD16/32-blocks Fc binding for 10 minutes in the dark to prevent unspecific reactions, and then antibodies and reagents, including Alexa Fluor 488 annexin V, propidium iodine, and PE-Cy5 anti-mouse CD3e, were added to resuspended cells in staining buffer and incubated for 15 minutes. After gating of lymphocytes by forward scattered light (FSC) and side scattered light (SSC), CD3-positive cells (T lymphocytes) were evaluated for the percentage of the normal and apoptotic cells using annexin V and propidium iodine staining, which are early and late apoptosis makers, respectively. T lymphocytes with early or late apoptotic markers were regarded apoptotic cells.

### 2.3. The Second Experimental Study

Here, we evaluated the effect of beta-blocker therapy on normal splenic T-lymphocyte numbers and apoptosis in the CLP model. Ten animals were allocated to 2 groups after the CLP procedure: control group which subcutaneously received 8 *μ*L/hour normal saline and esmolol group which administered 40 mg/kg esmolol bolus followed by subcutaneous infusion of 4 mg/kg/min (8 *μ*L/hour; *n* = 5 in each group). Esmolol was used in this study since this reagent has been widely used in the world and shown to have beneficial effects in the clinical randomized controlled study [13]. The dose of esmolol was determined in the preliminary study, in which this dose reduced heart rate by 20% in a healthy mouse. CLP was performed at the cecal quarter site proximal to the ileocecal valve (CLP-P) in both groups. After CLP (24 hours), the number of normal T lymphocytes per spleen and the percentage of apoptotic T lymphocytes per spleen were evaluated as in the first control study. PE-Cy7 anti-mouse CD4 and PE-Cy7 anti-mouse CD8a were also used to examine the rate of CD4+ helper and CD8+ natural killer T lymphocytes in total T lymphocytes in study 2.

### 2.4. Statistical Analyses

The 4 groups in study 1 were compared using the one-way ANOVA, followed by Tukey's test. The unpaired *t*-test was used to compare the control and esmolol groups in study 2. Results are presented as median (interquartile range) or mean ± SD, as appropriate. *p* < 0.05 was considered statistically significant.

## 3. Results

**Figure 1:**
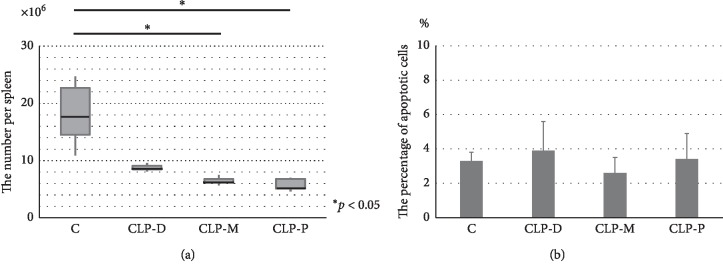
(a) Normal splenic T-lymphocyte numbers in sham operation mice (C) *n* = 5) and cecum ligation and puncture (CLP) mice at 3 different sites (CLP-D: cecal quarter site proximal to the ileocecal valve, CLP-M: midcecum, and CLP-P: cecal quarter site distal to the ileocecal valve; *n* = 5 per group). Normal T-lymphocyte numbers were decreased in proportion to CLP severity. (b) The percentage of T-lymphocyte apoptosis in 4 groups (C, CLP-D, CLP-M, and CLP-P). There were no significant differences in the percentage of apoptosis between the 4 groups. C: group C which received sham operation; CLP-D: group CLP-D which received CLP at the distal cecum; CLP-M: group CLP-M which received CLP at the midcecum; CLP-P: group CLP-P which received CLP at the proximal cecum.

As shown in Figure 1(a), the total normal splenic T-lymphocyte numbers per mouse significantly decreased in proportion to CLP severity. The differences between groups CLP-P and C, and CLP-M and C were statistically significant. The percentage of apoptosis, almost <5% in all 4 groups, did not differ significantly between the groups (Figure 1(b)).

**Figure 2:**
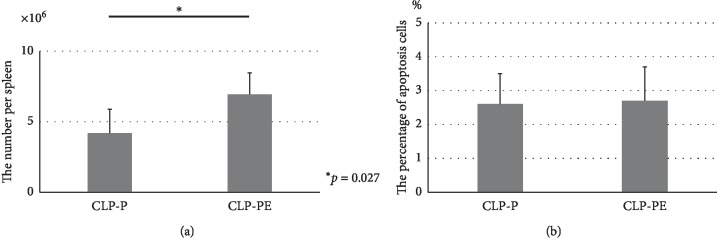
(a) Normal splenic T-lymphocyte numbers in CLP (proximal site) mice with (CLP-PE) or without (CLP-P) beta-blocker therapy. Beta-blocker therapy restored the normal T-lymphocyte numbers reduced by CLP. (b) The percentage of T-lymphocyte apoptosis in the CLP-P and CLP-PE groups. There was no significant difference in the percentage of apoptosis between the two groups. CLP-P: the group which underwent proximal site CLP; CLP-PE: the group which received beta-blocker therapy after proximal site CLP.

In the second experimental study, beta-blocker therapy preserved normal splenic T-lymphocyte numbers, reduced by CLP (Figure 2(a)), without affecting the percentage of apoptosis (Figure 2(b)). The percentages of helper and natural killer T lymphocytes were unaffected by esmolol administration (Table 1).

## 4. Discussion

In this study evaluating the beta-blocker therapy's effect on splenic T-cell lymphocytes in a mouse model of sepsis, continuous esmolol administration was found to preserve normal splenic T-lymphocyte numbers dramatically decreased by CLP (golden standard septic model) compared with the control group. This is the first study to present the beneficial effects of beta-blocker therapy in maintaining normal splenic T lymphocytes reduced in proportion to sepsis severity. Considering immunocompetent cell death, shown to contribute to worse sepsis prognosis [1–3], causes immune suppression, normal T-lymphocyte preservation by beta-blocker therapy represents an attractive therapeutic strategy for sepsis.

Sepsis is characterized as a high inflammatory response reflected by proinflammatory cytokine overproduction, followed by an immune paralysis phase. Although previous studies aimed at suppressing hyperinflammation at the early sepsis phase using large doses of steroid therapy [14] and antibodies against proinflammatory cytokines, including TNF-*α* [15], these clinical trials failed to show a beneficial effect (improved sepsis outcomes). Conversely, immunosuppression caused by lymphocyte apoptosis, following the early hyperinflammatory sepsis response, has been shown to contribute to poor sepsis outcome [1–3]. Circulating apoptotic lymphocytes, a main component of human septic shock immune dysfunction, are reportedly associated with poor prognosis [16]. Many experimental studies targeted lymphocyte apoptosis attenuation, based on the hypothesis that lymphocyte apoptosis prevention is a key factor to improving the prognosis of sepsis. Hotchkiss et al. demonstrated that z-VAD, a broad-spectrum inhibitor of caspases, which are key apoptosis-inducing enzymes, attenuated lymphocyte apoptosis and improved survival in a sepsis mouse model [4]. The same study showed that Bcl-2 (antiapoptotic protein) overexpression prevented lymphocyte apoptotic cell death in transgenic mice, leading to survival benefits. Another study by Schwulst et al. examined the effect of an agonistic antibody against CD40, a TNFR family member, on lymphocyte apoptosis and survival in experimental sepsis and showed that anti-CD40 treatment conferred protection against sepsis-induced lymphocyte apoptosis via Bcl-x_L_ (antiapoptotic protein) upregulation and improved survival in sepsis [17]. Furthermore, while other therapies targeting lymphocyte apoptosis, including peptide-mediated Akt activation and extracellular-regulated kinase signaling, which have antiapoptotic properties, and the siRNA of cell death proteins, Bim and PUMA, have been shown to protect lymphocytes from cell death [5, 6], few studies have evaluated the benefits of lymphocyte apoptosis-targeting therapies in clinical situations. This is likely because the safety of these therapies, including caspase inhibitor, cell-permeable peptide, and siRNA, has not been proven in humans. Thus, interventions or drugs already in clinical use with an ability to prevent sepsis-induced lymphocyte cell death are quite attractive.

Beta-blocker therapy has been widely conducted in the ICU, even though their administration to sepsis patients remains unapproved. Previous studies have described a relationship between catecholamine stimulation and lymphocyte apoptosis induction [8], preventive effect of beta-blocker against splenocyte apoptosis in a hemorrhage shock model [18], protective effects of beta-blocker against staurosporine-induced apoptosis in SH-SY5Y neuroblastoma cells [19], and lymphocyte function modulated by catecholamine stimulation [20]. These results indicate that the sympathetic nerve system is associated with lymphocyte function, apoptosis, and cell death regulation. In this study, the beta-blocker esmolol, preserved normal splenic T-lymphocyte numbers, reduced in proportion to sepsis severity, but the mechanisms were not examined. However, the mechanisms of esmolol-induced attenuation of normal T-lymphocyte reduction in septic models could be considered. Previous studies have shown that beta-blockers suppress inflammatory cytokine overproduction [9, 11], among which the proinflammatory cytokine TNF-*α,* has been proven to induce lymphocyte cell death via the extrinsic apoptotic pathway [7] in sepsis. The suppressive effect of beta-blockers against cytokine production could be one possible mechanism. Another possible mechanism is that beta adrenergic stimulation itself has been shown to be associated with lymphocytes apoptosis and cell death induction. An experimental study demonstrated that dopamine and dobutamine stimulation induced apoptosis of peripheral blood lymphocytes purified from blood samples of normal healthy volunteers, but was attenuated by propranolol pretreatment [8]. Considering that beta adrenergic receptors exist on the surface of lymphocytes, their stimulation may regulate lymphocyte cell death.

There are several limitations to interpreting the data herein. Firstly, although we detected only small percentage of T lymphocyte apoptosis, contrary to our expectation, the number of normal T lymphocytes was reduced extremely instead in this model. Lymphocyte apoptosis was evaluated 24 hours after CLP procedure following the previous study which examined lymphocyte apoptosis in the same model [4]. Although the reason for this discrepancy between our study and the previous study is unknown, we believe that the reduced normal T lymphocyte number reflects the severity of immunocompromised condition and restored normal T lymphocytes by esmolol administration represents the beneficial effects of beta-blocker therapy. Secondly, since esmolol's effect on mortality was unexamined in this study, it is unknown whether normal T-lymphocyte number preservation in the spleen has survival benefits. However, esmolol treatment restored the normal T-lymphocyte numbers reduced by CLP-P (CLP, cecal proximal site) to the same level as CLP-M (CLP, midcecum). Considering that the prognosis of CLP-P is worse than that of CLP-M, esmolol is likely to improve the prognosis of the CLP septic model. Thirdly, the mechanisms by which beta-blocker, esmolol, provided a restoration of normal splenic T lymphocytes reduced by septic insult were not examined. As described earlier, there are some speculations that should be considered as possible mechanisms. Although mechanistic study is for future research, the aim of this study, to examine beta-blocker effect on splenic T lymphocytes, has been accomplished. Fourthly, after CLP, blood pressure and heart rate, whose reduction could have affected the results of this study, were unmonitored. As mentioned in Section 2, in the preliminary study, the dose of esmolol used in this study reduced heart rate by 20% in healthy mouse. As esmolol dose reducing heart rate by 20% in the healthy mouse did not reduce blood pressure in the previous study showing cardioprotective effects in the septic rat model [9], we can therefore assume that esmolol did not reduce the blood pressure of the present study's model. Fifthly, we did not measure inflammatory cytokine levels, which have been shown to play an important role in modulating immunological function. Finally, no antibiotic administration was not clinically relevant in this model. However, we could evaluate the immunomodulatory effects of esmolol without the interference of antibiotics against immunological function.

## 5. Conclusions

In this study, esmolol administration restored normal splenic T-lymphocyte numbers, reduced in proportion to sepsis severity. Since septic insult-induced immunodeficiency has been shown to contribute to the worse prognosis of septic patients, the ability of beta-blockers to preserve normal T lymphocytes may improve the prognosis of sepsis. We should elucidate the mechanisms in which esmolol preserved the number of normal T lymphocytes and examine the effect of beta-blocker therapy on immunological function in human septic patients as the next step to enable clinical application.

## Figures and Tables

**Table 1:** The percentage of CD4+ and CD8+ cells in control and esmolol groups.

	Control	Esmolol	*p* value
CD4+ cells (%)	55.6 ± 1.3	52.6 ± 3.4	0.102
CD8+ cells (%)	39.2 ± 0.3	41.6 ± 2.7	0.077

## Data Availability

The data used to support the findings of this study are included within the manuscript.
